# A Case of Phlegmasia Cerulea Dolens

**DOI:** 10.7759/cureus.10187

**Published:** 2020-09-01

**Authors:** Davin Barnett, John Casey

**Affiliations:** 1 Emergency Medicine, OhioHealth Doctors Hospital, Columbus, USA

**Keywords:** phlegmasia, cerulea, dolens

## Abstract

Phlegmasia cerulea dolens (PCD) is a rare presenting condition that carries a high mortality and morbidity risk. It is a cannot miss diagnosis with potentially deadly sequelae, and we highlight the importance of a thorough physical examination and initiation of empiric treatment to help prevent such.

## Introduction

Phlegmasia cerulea dolens (PCD) is a rare presenting condition that carries a high mortality and morbidity risk [1]. We present a case of a classic presentation of this condition and emphasize the importance of a thorough physical examination with a comparison of extremities when assessing a patient as well as early therapy initiation. Prompt recognition and initiation of empiric treatment are essential to prevent life-threatening sequelae. 

## Case presentation

**Figure 1:**
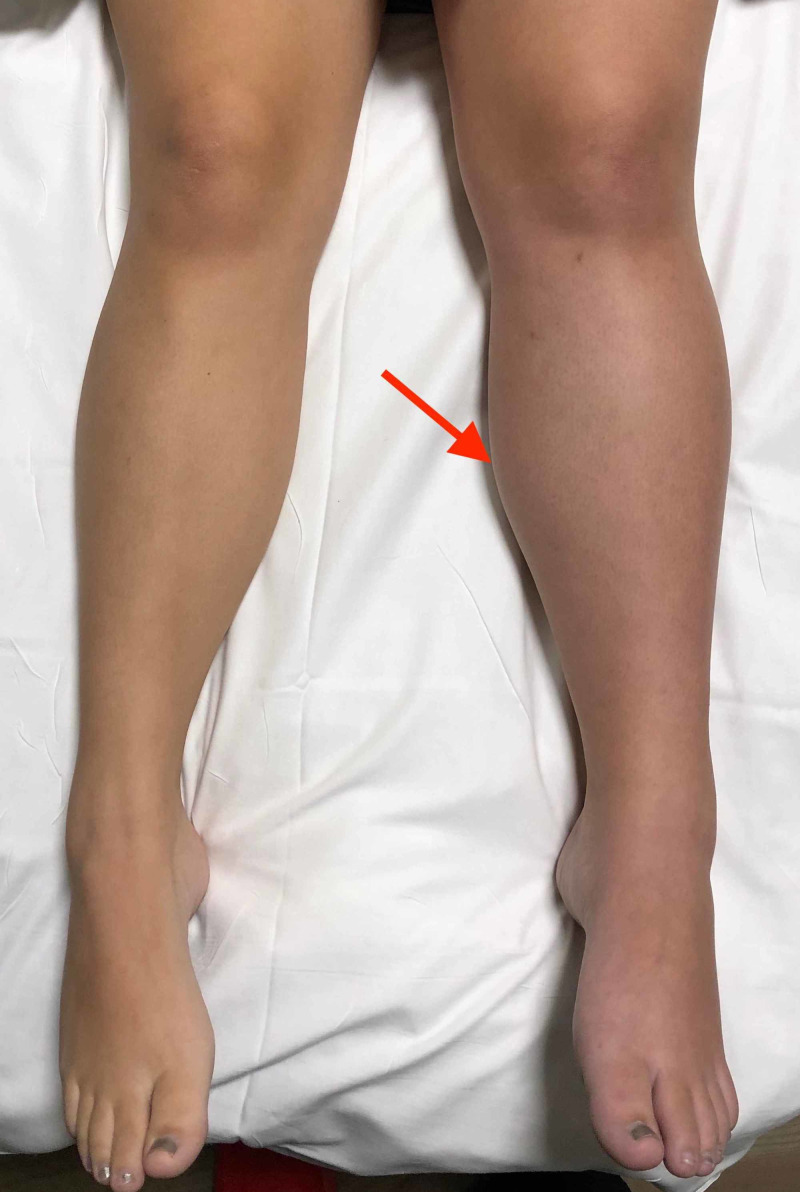
Comparison of bilateral lower extremities. Solid red arrow demonstrating left leg edema, plethora, cyanosis.

A 28-year-old female with a history of factor V Leiden thrombophilia not on anticoagulation presented to the emergency department (ED) for left lower extremity pain and difficulty ambulating that had worsened over the previous three days. The patient was three-weeks postpartum from an uncomplicated spontaneous vaginal delivery. There was no prior history of deep venous thrombosis or pulmonary embolism. She denied trauma or injury to the area. Initial musculoskeletal examination of the fully clothed patient indicated mild tenderness upon palpation of the left leg but was otherwise unremarkable. The patient ambulated into the emergency department with a slight limp. The patient repeatedly minimized her presenting symptoms. Completion of the physical examination, conducted with the patient fully gowned, revealed a blue-grey discolored/mottled left lower extremity, diffusely tender to palpation. Dorsalis pedis and posterior tibial pulses were both palpable in the affected extremity. The appearance of the left lower extremity is represented below (Figure 1). Ultrasound of the leg showed an acute deep vein thrombosis with extensive clot burden in the left external iliac, common femoral, femoral, popliteal, gastrocnemius, and posterior tibial veins. Acute superficial vein thrombosis was also present in the small saphenous vein and the great saphenous vein at the junction to the knee.

**Figure 2:**
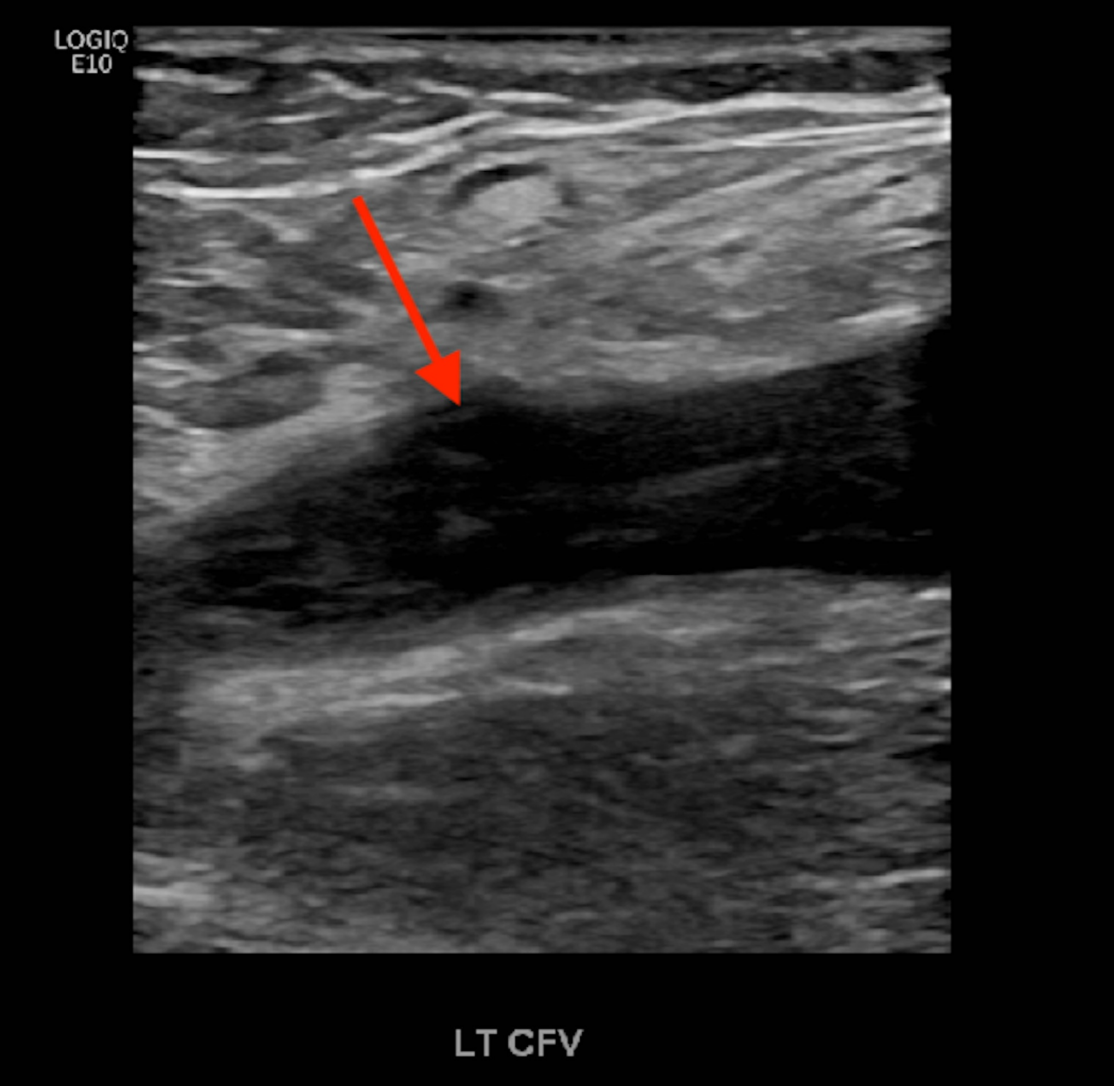
Ultrasound demonstrating echogenic (thrombosed) blood within the common femoral vein (red arrow).

Figure 2 shows the ultrasound demonstrating echogenic (thrombosed) blood within the common femoral vein (red arrow).

**Figure 3:**
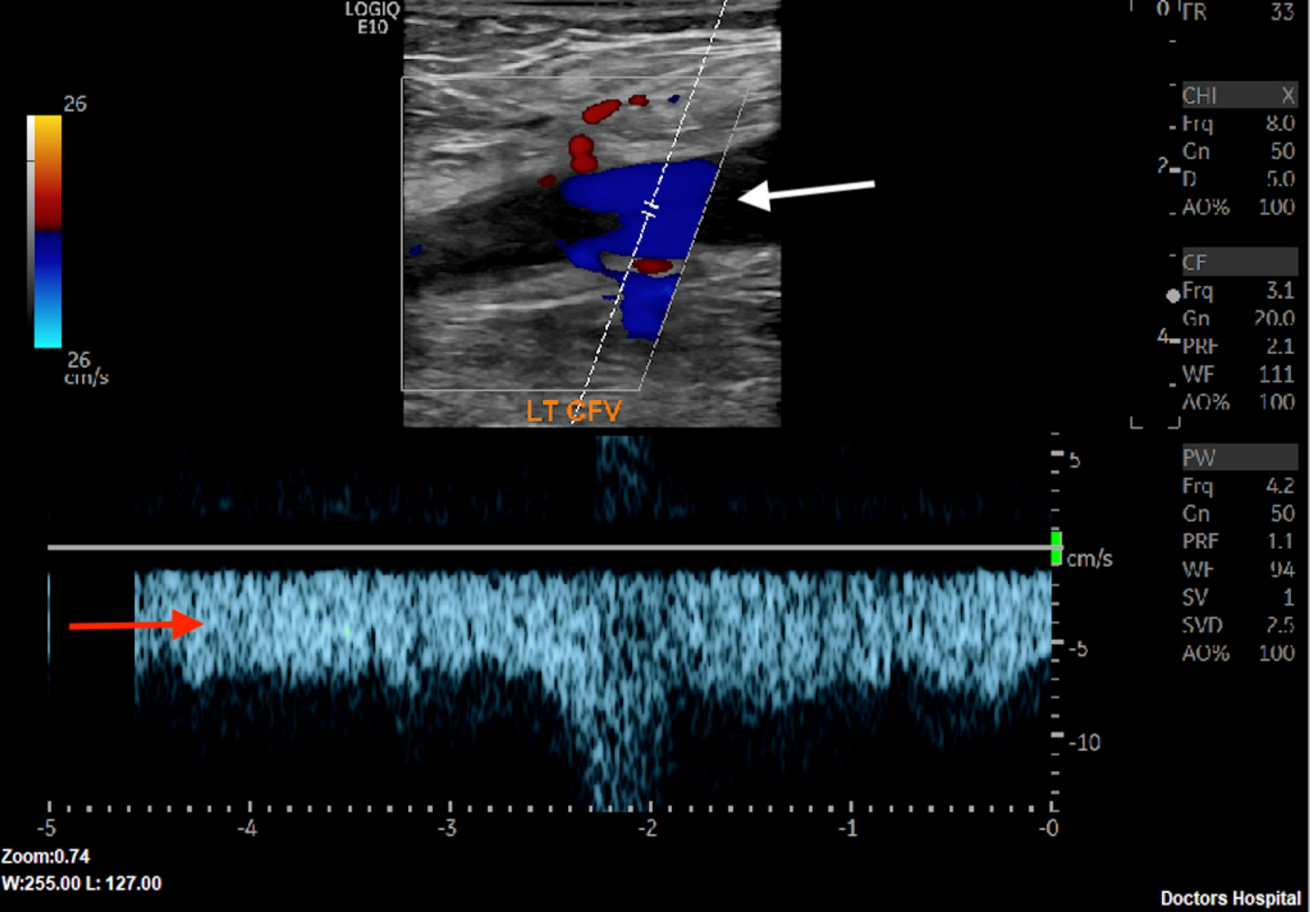
Ultrasound image indicates Doppler is of focus (white arrow) in the left CFV. Graph at bottom (red arrow) demonstrating monophonic Doppler signal and lack of venous flow suggesting obstruction by clot burden. CFV: common femoral vein.

Ultrasound with color flow Doppler demonstrating monophasic Doppler with lack of venous flow beyond clot burden is shown in Figure 3.

## Discussion

With our patient, anticoagulation with heparin was initiated in the ED prior to imaging and the patient was admitted with vascular surgery consultation. Vascular surgery recommended thrombectomy due to clot burden and concerns for post-thrombotic syndrome. However, the patient refused invasive interventions. Hematology was consulted with recommendations for Lovenox to warfarin bridge and anticoagulation for three months. On day 3 in the hospital, the patient developed acute dyspnea, tachypnea, and fever. The computed tomography pulmonary angiogram revealed pulmonary embolism. The patient continued to refuse all invasive procedures, including IR thrombectomy. Anticoagulation was continued with some symptom improvement and was ultimately discharged on day 5 from the hospital with warfarin and close follow-up. The patient was subsequently diagnosed with May-Thurner syndrome.

Phlegmasia cerulea dolens, a massive proximal venous thrombosis of the lower extremity, is a rare entity that carries a mortality rate of 20-40% [1-4]. It is a disease process necessitating a thorough physical examination, rapid diagnosis, and initiation of treatment. According to Perkins et al., PCD is characterized by an extensive venous thrombus that obstructs venous drainage leading to fluid sequestration and edema of surrounding tissues [3]. Risk factors include malignancy, hypercoagulable states, contraceptives, trauma, venous stasis, May-Thurner anatomy, inferior vena cava filters, and surgeries [5]. Complications include venous gangrene, compartment syndrome, and arterial compromise. Sequelae of this disease include venous gangrene 40-60%, pulmonary embolism 50%, and amputation 10-25% [3].

Early recognition of PCD and prompt initiation of anticoagulation are critical to minimize potentially fatal sequela of this condition. This case illustrates the importance of the fundamental tasks of having patients disrobe for a physical examination, comparing extremities, and investigating complaints even when they are minimized by the patient.

## Conclusions

PCD is a rare disease with significant morbidity and mortality. Early diagnosis and treatment are necessary to prevent severe complications such as limb ischemia or massive pulmonary embolism. As in this case, prompt recognition and initiation of empiric treatment are essential to prevent life-threatening sequelae.
